# Pathogenic Copy Number Variations Involved in the Genetic Etiology of Syndromic and Non-Syndromic Intellectual Disability—Data from a Romanian Cohort

**DOI:** 10.3390/diagnostics12123137

**Published:** 2022-12-12

**Authors:** Ioana Streață, Alexandru Caramizaru, Anca-Lelia Riza, Simona Șerban-Sosoi, Andrei Pîrvu, Monica-Laura Cara, Mihai-Gabriel Cucu, Amelia Mihaela Dobrescu, Ro-NMCA-ID Group, CExBR Pediatric Neurology Obregia Group, CExBR Pediatric Neurology “V. Gomoiu” Hospital Group, Elena-Silvia Shelby, Adriana Albeanu, Florin Burada, Mihai Ioana

**Affiliations:** 1 Regional Centre of Medical Genetics Dolj, Emergency County Hospital Craiova, 200642 Craiova, Romania; 2 Laboratory of Human Genomics, University of Medicine and Pharmacy of Craiova, 200638 Craiova, Romania; 3 Doctoral School, University of Medicine and Pharmacy of Craiova, 200349 Craiova, Romania; 4 Department of Public Health, University of Medicine and Pharmacy of Craiova, 200638 Craiova, Romania; 5 The Ro-NMCA-ID (RoNetwork Multiple Congenital Abnormalities with ID) Member of European Reference Network on Rare Congenital Malformations and Rare Intellectual Disability (ERN-ITHACA) [EU Framework Partnership Agreement ID: 3HP-HP-FPA ERN-01-2016/739516], 400011 Timisoara, Romania; 6 Pediatric Neurology Rare Disease Expertise Center Obregia, Sos. Berceni nr. 10, Sector 4, 041914 Bucuresti, Romania; 7 Center for Expertise in Rare Diseases in Pediatric Neurology, Pediatric Neurology Clinical Department, Children’s Clinical Hospital “Dr. Victor Gomoiu”, 022102 Bucharest, Romania; 8 National University Center for Children’s Neurorehabilitation “Dr. Nicolae Robănescu”, 44 Dumitru Mincă Street, District 4, 041408 Bucharest, Romania; 9 Department of Pediatric Neurology, Clinical Emergency Children Hospital Brasov, Nicopole Street No. 45, 500063 Brasov, Romania

**Keywords:** intellectual disability, global developmental delay, microdeletion/microduplication, CNV, chromosome microarray analysis

## Abstract

The investigation of unexplained global developmental delay (GDD)/intellectual disability (ID) is challenging. In low resource settings, patients may not follow a standardized diagnostic process that makes use of the benefits of advanced technologies. Our study aims to explore the contribution of chromosome microarray analysis (CMA) in identifying the genetic etiology of GDD/ID. A total of 371 Romanian patients with syndromic or non-syndromic GDD/ID, without epilepsy, were routinely evaluated in tertiary clinics. A total of 234 males (63.07%) and 137 (36.93%) females, with ages ranging from 6 months to 40 years (median age of 5.5 years), were referred for genetic diagnosis between 2015 and 2022; testing options included CMA and/or karyotyping. Agilent Technologies and Oxford Gene Technology CMA workflows were used. Pathogenic/likely pathogenic copy number variations (pCNVs) were identified in 79 patients (21.29%). Diagnosis yield was comparable between mild ID (17.05%, 22/129) and moderate/severe ID 23.55% (57/242). Higher rates were found in cases where facial dysmorphism (22.97%, 71/309), autism spectrum disorder (ASD) (19.11%, 26/136) and finger anomalies (20%, 27/96) were associated with GDD/ID. GDD/ID plus multiple congenital anomalies (MCA) account for the highest detection rates at 27.42% (17/62). pCNVs represent a significant proportion of the genetic causes of GDD/ID. Our study confirms the utility of CMA in assessing GDD/ID with an uncertain etiology, especially in patients with associated comorbidities.

## 1. Introduction

Neurodevelopmental disorders (ND), including global developmental delay (GDD)/intellectual disability (ID) and/or autism spectrum disorders (ASD), affect 1–3% of the world’s population [1]. In developed countries, severe ID is reported in 2.5 to 5 in 1000 children, while mild ID has a higher prevalence, especially among children with low socioeconomic status [2].

The cited causes of GDD/ID include prenatal and perinatal infections or trauma, genetic abnormalities, environmental factors, metabolic anomalies, nutritional deficits and toxic exposure, but in 75% of cases, the etiology is unknown [3]. Various genetic causes can lead to GDD/ID; the most frequent is Down syndrome [4], while Fragile X syndrome is the most common inherited cause [5]. Genetic abnormalities involved include aneuploidies, copy number variations (CNVs), tandem repeats, indels and short variation [1,6].

The clinical and genetic heterogeneity of GDD/ID hampers the genetic diagnosis success rates. However, technological advances in genetic testing through array comparative genomic hybridization (aCGH) and next-generation sequencing (NGS) have provided insight into the genetic underlying factors of GDD/ID. In 2010, the International Standards for Cytogenomic Arrays (ISCA) Consortium recommended molecular karyotyping by chromosome microarray analysis (CMA)—aCGH and SNP-aCGH (single nucleotide polymorphism-aCGH)—as the first-tier cytogenetic diagnostic test in the investigation of patients with GDD/ID, ASD and multiple congenital anomalies (MCA) [7]. Since then, a growing number of publications have reported varying diagnostic yields of CMA in cohorts of patients with GDD/ID from different regions of the globe—a worldwide average rate of 10% to 25% in recent years [8,9,10,11,12,13,14,15,16,17,18,19,20,21,22,23,24].

Romanian data is scarce. To our knowledge, there is a single publication about CMA in a small cohort of 36 patients with GDD/ID and obesity from a north western region of our country [25].

Our study aims thus to present the results of aCGH and karyotype testing from a Romanian cohort of 371 patients with GDD/ID, contributing to the existing reports on the utility of aCGH in GDD/ID diagnosis and the involvement of various chromosomal regions in the etiology of this complex pathology.

## 2. Materials and Methods

### 2.1. Patient Inclusion and Evaluation

This study includes 371 patients evaluated for GDD and/or ID in pediatric, child neurology or medical genetics departments from different regions of Romania. They had been referred to the Regional Centre for Medical Genetics (CRGM), Dolj, Craiova for genetic testing between 2015 and 2022. A total of 234 of the patients were boys (63.07%) and 137 (36.93%) were girls, with ages ranging from 6 months to 40 years (median age was 5.5 years) at their first medical assessment. 

Inclusion criteria to the current study were the presence of GDD in children <5 years old or ID in children over 5 years old [26], and the absence of epilepsy. Epilepsy can determine ID; a different diagnostic approach may be better suited [27].

Most of the patients showed syndromic involvement, with the presence of dysmorphic features and/or various malformations. 

Clinical evaluations, including personal history, psychomotor and behavioral development, ID severity, the presence of dysmorphic features, neuroimaging and EEG studies, were obtained from referring clinicians, neurologists or pediatric neurologists.

ID severity was classified as mild, moderate, severe or profound by DSM-5 (*Diagnostic and Statistical Manual of Mental Disorders, 5th Edition*) criteria, following the evaluation of conceptual, social and practical impairment.

Ethical approval for the study was granted by the local research ethics committees of the involved institutions. A written informed consent form was signed by the parents or legal guardians of the patients.

### 2.2. Genetic Testing

Resource limitations at times dictated the first intention genetic test, despite recommendations. For 169 patients, karyotyping was used as the first choice. All 371 cases, irrespective of karyotyping results, were run though CMA as soon as it became available.

High-resolution aCGH was performed using testing options from Agilent Technologies, Santa Clara, CA, USA: Agilent SurePrint G3 CGH ISCA v2 8 × 60K (141 patients), 4 × 180K (37 patients), and Oxford Gene Technology Operations Ltd.: CytoSure ISCA V2 CGH 8 × 60K microarrays (193 patients), following the protocols provided by the manufacturer [28]. A feature extraction program was used to obtain post-hybridization data. Subsequent analysis was performed with the recommended software: CytoGenomics software from Agilent and Cytosure Interpret Software from OGT, respectively.

CMA, standard karyotyping and/or multiplex ligation-dependent probe amplification (MLPA) were used to confirm the CMA findings and perform segregation analysis where commercial kits to cover the region of interest were available, and/or parents consented.

### 2.3. Variant Interpretation and Reporting

Quality criteria included a coverage of at least 4 hybridized probes with a minimum average ratio of 0.5 and a software reported *p* value of less than 0.05. Deletions do not require size cut-offs [29]; only duplications larger than 250 kb were reported, as it is more difficult to assign a pathogenic role to copy number gains. Variants with >50% overlap with the CNVs detected in healthy individuals in the Database of Genomic Variants (DGV) and those without gene content were eliminated from the analysis. Reported genomic regions use GRCh37/hg19 for reference purposes.

The CNVs filtered as previously described were further classified based on ACMG (American College of Medical Genetics) guidelines [30] for the reporting of postnatal CNVs using a 5-tier classification: pathogenic, likely pathogenic, uncertain significance (VUS), likely benign and benign. Public databases including Online Mendelian Inheritance in Man (OMIM) [31], ClinVar [32], ClinGen Dosage Sensitivity Map [33], ISCA [34], Clinical Genomic Database (CGD) [35], and DECIPHER [36], as well as the published literature [37], were queried to identify evidence supporting the pathogenic characteristics of CNVs.

For the current study, only clinically relevant findings, pathogenic/likely pathogenic CNVs (pCNVs), are reported. Incidental findings were not disclosed, as per patient consent.

Univariate analysis (odds ratio OR, with confidence intervals CI) indicated predictive phenotypes for a higher diagnostic yield—a higher chance to have a pCNV—in our cohort with unexplained GDD/ID. Data were analyzed using SPSS Statistics for Windows, Version 22.0 (IBM, Armonk, NY, USA: IBM Corp).

## 3. Results

### 3.1. Diagnosis Rate

**Figure 1:**
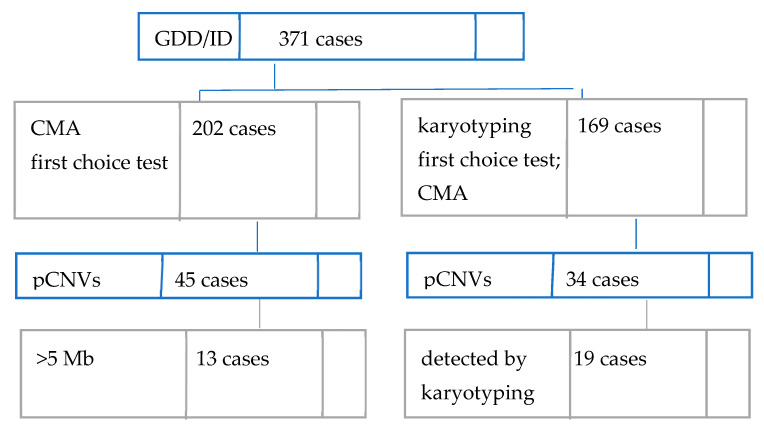
Diagnostic workflow and findings in our cohort.

In our cohort of 371 Romanian individuals with unexplained GDD/ID with syndromic or non-syndromic presentation, 79 patients were found to carry at least one rare exonic pCNV, as seen in Figure 1 below. This places the overall diagnostic yield of aCGH in the current study at 21.29%.

Conventional karyotyping had been performed prior to CMA for 169 patients. Performing CMA on the 19 cases where karyotyping identified pCNVs was requested to better define the regions involved. The diagnostic rate for karyotyping is 11.24% (19/169). pCNVs larger than 5 Mb could have been detected by classic karyotyping. If we are to assume the same accuracy as CMA for >5 Mb deletions/duplications, then, in our study, the karyotyping diagnosis rate would become 8.62% (32/371).

### 3.2. Genetic Findings

Detailed findings are described in Table 1: absolute coordinates and the size of the pCNVs, the number of OMIM genes reported, the major phenotypic characteristics, the diagnosed syndrome and *de novo* status.

pCNVs findings were not significantly different between males and females: 49 (20.94%) male patients and 30 (21.90%) female patients. Five of our cases had two pCNVs concurrently.

The average size of pCNVs ranged from 0.07 Mb to 55.02 Mb with a median of 0.65 Mb. Similar to previously published studies, the absolute size for losses was generally smaller than that of gains: a median size of 5.31 Mb compared to 8.44 Mb, respectively.

pCNVs were generally equally distributed in almost all chromosomes; CNVs were not identified in chromosomes 4, 6 and Y in our cohort. An enrichment of pCNVs was found in chromosomes 22 and 15, mainly due to a few common syndromes identified in our cohorts—Prader–Willi/Angelman syndrome (3 patients), 15q11.2 duplication syndrome (6 patients), 22q11.2 deletion syndrome (1 patient) and 22q11.2 duplication syndrome (3 patients). The chromosomes with less frequent CNVs (below 3 cases) were chromosomes 5, 10, 11, 12, 14, 19, 20 and 21.

As Table 1 describes, in these 79 cases with clinically relevant findings, we found 84 rare exonic pCNVs—35 gains and 49 losses, assigned as follows:

- pCNVs overlapping with known genomic disorders were found in 34/84 (40.48%) of CNVs, out of which 15 were gains and 19 were losses. These are associated with known microduplication or deletion syndromes, allowing genetic diagnosis for 9.16% of the patients of this study.
- pCNVs not associated with any known syndrome, but already reported in the literature, were found in 50/84 (59.52%) of CNVs, with 20 gains and 30 losses.

Where possible, we aimed to evaluate the *de novo* status and/or segregation patterns of the detected genetic anomalies in trios of mother–father–affected individual; in 2 cases siblings were also analyzed. We classified 35 aberrations as *de novo*; one of these was also present in the proband’s sibling, a case with an otherwise similar clinical phenotype (Table 1, cases #220 and #225). A total of 49 CNVs were reported to be of unknown inheritance where testing results were not available at the time of the article being published.

### 3.3. Clinical Findings

The major phenotypic characteristics of the cases are reported in Table 1. The cohort characteristics are summarized in Table 2. Most patients presented additional features, including ASD, MCA, psychiatric or behavioral issues, cranio-facial dysmorphism, skeletal and muscular anomalies, and variations in height or body weight. The relationship between these and GDD/ID remains unclear. Many cases presented syndromic features, as can be concluded by the high presence of MCA and atypical facial appearance.

Table 2 summarizes the cohort characteristics and provides statistical calculations for each clinical feature in order to test if it is a predictive phenotype for a higher diagnostic yield in our cohort with unexplained GDD/ID. All the patients in our study had GDD at the time of the study or at an earlier age, with 89.22% considered intellectually disabled. Divided into subgroups based on ID severity, the group of mild ID had a higher diagnostic yield (17.05%) compared with the moderate, severe and profound ID group (23.55%).

Facial dysmorphisms, though mostly minor findings, were reported in 83.28% of cases, microcephaly or macrocephaly in 37.46%, congenital anomalies in 16.71%, ASD in 36.65%, ADHD in 20.21% and speech/language delay in 64.15%. Other phenotypes had lower frequencies. Most patients had more than one associated feature.

We found associations between positive findings and clinical features with: hearing impairment (OR = 2.58), dysmorphic facial features (OR = 2.01), fingers abnormalities (OR = 1.68), congenital anomalies (OR = 1.50), ADHD (OR = 1.21) or psychiatric disturbance (OR = 0.48). There was no significant higher diagnostic yield by CMA for the other phenotypes.

## 4. Discussion

### 4.1. Diagnosis Rate and Choice of Test

The diagnostic yield that CMA provides is reported to be between 10 and 25%, higher than karyotype testing, as also shown by our findings. A large cohort study has reported 118 rare *de novo* CNVs associated with ID [38]. Further analysis of the respective regions identified 10 genes for which a loss of function could lead to ID. [39] In a group of 342 children with unexplained GDD or ID, aCGH detected pCNVs in 13.2% of the patients [40]. In Table 3, we present the diagnosis yield from recent microarray studies on European cohorts with GDD/ID.

As it stands, CMA remains the first choice of diagnostic tool for the detection of causative CNVs in human diseases at present in many health systems. Despite this recommendation, in low-resource settings conventional karyotyping is still a widely used genetic test in clinical practice. Although CMA has proved its role in identifying genetic causes of neurodevelopmental disorders, in Romania conventional karyotyping is still the predominant genetic test in clinical practice. There are only a few publications of CMA in cohorts of ND patients. Diana Miclea et al. analyzed 36 patients with GDD/ID and obesity from northern Romania between 2015 and 2017, using the iScan System (Illumina, San Diego, CA, USA) with a diagnostic yield of 33.3% [25]. Our study analyzed a cohort of 371 patients from Romania, who underwent microarray testing for diagnostic purposes between 2015 and 2022. This is among the first CMA studies of Romanian patients with unexplained GDD/ID and additional comorbidities. In the current study, a total of 84 pathogenic changes were detected among 79 patients with syndromic GDD/ID (21.29%). Our diagnostic rates are in line with previous reports from multicenter studies [7,23,24].

CMA diagnostic yield reported in the literature may vary largely, being the subject of different selection criteria for patient inclusion, CNV classification and/or the inclusion of control groups, as well as the preliminary exclusion of large genomic imbalances [42]. In our study, we did not apply strict selection criteria—the presence of GDD/ID associated, or not, with MCA, ASD, or dysmorphic features. Patients with seizures or epilepsy were not included in this cohort.

Existing publications recommend a minimum resolution of 200–400 kb for postnatal analyses [7,45,46]. CMA was performed in our center using several types of DNA microarrays (180K and 60K), without any notable difference in their diagnostic reliability. Although 180K platforms offer a three-fold higher resolution, we also find that both 60K and 180K microarray platforms comply the existing requirements regarding resolution, similar with previous reports [22].

The detection of submicroscopic gains and losses of genetic material known as CNVs is the major advantage of CMA over G-banded karyotyping. Due to the higher resolution and whole genome coverage CMA offers, it can precisely identify the chromosomal breakpoints, the size, and gene content of CNVs. This impacts identification of clinically relevant microdeletion/duplication in the context of the patient’s phenotype. In contrast to aCGH, SNP-aCGH allows the detection of triploidy, low-level mosaicism, loss of heterozygosity (LOH) and uniparental disomy (UD).

CMA is not only a highly reliable confirmatory test of chromosomal aberrations detected through conventional G-banding karyotyping, but it is also able to specify the size and gene content. In the subgroup of patients with karyotyping offered as a first-choice test, all identified microdeletion and microduplication syndromes had been already reported, whereas the 13 additional ones identified by CMA were novel.

We need to emphasize that a complete genetic diagnosis may require complementary methods, e.g., both CMA and karyotyping [47,48]. CMA did not make karyotyping obsolete. Balanced rearrangements remain undetectable regardless of the type of array used.

For patients #315 and #328, we used a combined strategy including G-banding karyotyping and CMA. Conventional karyotyping offered the global and complex analysis of chromosomal organization and CMA confirmed and characterized the chromosomal rearrangements at molecular level. This stands as proof that CMA cannot entirely replace the standard conventional G-banding karyotyping due to its inability to detect balanced chromosomal rearrangements or to specify gains of DNA sequences in the karyotype.

Standardization of the diagnostic process in GDD/ID with unexplained etiology is an endeavor that has to continually adapt to diagnostic challenges and technical solutions. G-banding karyotyping has been frequently utilized, but slowly transitioned to being an adjunct to microarray technology [49]. Best practices for the first-line assessment of unexplained GDD recommend microarray as the first-line means of genetic investigation [50]. There is a boon of studies on CMA use in GDD/ID associated, or not, with ASD, MCA and facial dysmorphism [7,24,51,52,53]. The most recent position statement maintains the recommendation, adding whole-exome (WES) or -genome sequencing (WGS) to increase identification of causal variants in up to 40% of patients with severe ID, as they can detect most CNVs and additional gene variations [54,55,56].

### 4.2. Cohort Findings

The recommended criteria to establish CNV pathogenicity include chromosome rearrangement size, gene content, inheritance pattern and making use information from databases and the relevant literature to check overlap with dosage sensitive regions [7,57].

The pCNVs identified in our cohort were typically very large, with a mean size of 2.46 Mb (median: 0.65 Mb), and most of them contained multiple genes. Some of the benign CNVs situated in gene-poor regions, such as those close to centromeres, unreported here, were very large.

In our cohort, in submicroscopic pCNVs, we observed more than a 1.4-fold higher frequency of microdeletions than microduplications (58.33% vs. 41.67%). It is well known that due to the content of dosage sensitive haplo-insufficient genes, microdeletions have a higher pathogenicity than their reciprocal microduplications [44,58,59]. A clinical interpretation of microduplication requires the assessment of their *de novo* status, gene content analysis and public databases of CNV interrogation [60]. Usually, microduplications larger than 1 Mb are expected to be likely pathogenic. In our cohort, we observed that CNVs larger than 1 Mb are more likely to have a pathogenic/likely pathogenic effect on the phenotype. We detected a total of 12 CNVs smaller than 500 kb with a defined clinical impact: 25% of them were *de novo* (3/12) and 75% of them (9/12) lacked this assessment.

Similar to previous publications [19,61,62], chromosomal imbalances involving 15q11.2, 16p11.2 and 22q11.2 loci were the most common findings in our cohort, with 6, 4 and 3 CNVs, respectively. These CNVs are characterized by incomplete penetrance and have been associated with a wide variety of phenotypes, including GDD/ID, ASD, psychiatric disorders, and epilepsy [63,64,65]. In these cases, clinical interpretation and family genetic counseling is challenging, especially so if an inheritance assessment of these chromosomal imbalances is not possible or they are inherited from a healthy parent [66,67]. In our group, parental testing could be performed for all patients within this category: all of them presented *de novo* CNVs.

In our cohort, we identified three cases of 15q11.2 microdeletion. The literature reports 15q11.2 microdeletion as one of the most common chromosomal abnormalities involved in the pathogenesis of ASD [44,68]. We also identified two cases of proximal 16p11.2 microdeletion (all of them *de novo*). This region is characterized by variable penetrance. Rosenfeld et al. reported in 2013 that the 16p11.2 microdeletion has a penetrance of 46.8%, which is compatible with its powerful adverse impact on the phenotype [64]. In our study, all the CNVs detected in these regions were *de novo*, having a higher general penetrance. The penetrance analysis also supports the role of other CNVs detected in our cohort (proximal 1q21.1 microduplication, 15q11.2 microdeletion), with low penetrance (<20%) as “risk” or “susceptibility loci” in the pathogenesis of GDD/ID, ASD and MCA.

The implementation of CMA in genetic testing practice has rapidly increased the diagnostic yield of idiopathic GDD/ID associated with MCA, ASD and/or facial dysmorphism by allowing the identification of novel submicroscopic rearrangements involved in the pathogenesis of these clinical phenotypes. This is a critical and challenging point in CMA data interpretation.

Based on the clinical data, the most frequently reported phenotypes are also the main reasons of referral: GDD/ID, MCA and/or dysmorphia, and ASD (Table 2). For instance, congenital anomalies, along with facial dysmorphisms, were reported in more than 16.71% of our cohort. Univariate analysis showed a significant association for the presence of pCNVs with dysmorphic facial features, psychiatric disorders, MCA and finger anomalies. Furthermore, secondary phenotypes, ASD and speech/language delay, fingers abnormalities and hearing impairment, were shown to be associated with higher findings of pCNVs in our patients with GDD/ID. However, larger sample sizes would be crucial to confirming these findings.

### 4.3. Limitations and Perspectives

There are several limitations to our current study, that we acknowledge, and have tried to tackle to the best of our abilities: (i) our study’s sample size is relatively small; (ii) subjective assessment of some clinical features cannot be excluded; (iii) the lack of validation by other molecular genetic assays for some of the patients; (iv) inheritance status is missing for the interpretation of rare variants; (v) balanced abnormalities, small-scale mutations and low-level mosaicism cannot be detected by CMA and need to be further evaluated; (vi) sequencing can to look for additional variation that may explain this complex pathology.

CMA represents a standard method in the genetic diagnostic multistep algorithm, serving as a first-tier test, confirmatory test or test following conventional G-banding karyotyping as well. Despite the good diagnostic yield reported, most of our cases remain undiagnosed and more complex genetic tests based on NGS methods are required to identify the genetic etiology. This we recognize as a limitation of the genetic evaluation we were able to provide, which we hope to address in future diagnostic workflows. A lack of sequence information does not enable us to identify relevant causal genetic variations or refine the diagnosis by identifying recessive conditions revealed by deletions in our cohort, for instance.

## 5. Conclusions

There are only a few publications on CMA testing in cohorts of GDD/ID patients with associated comorbidities from East European countries. Our study is among the first CMA evaluation reports of a Romanian patient cohort with unexplained GDD/ID and additional comorbidities.

The reported diagnostic rate for CMA in this study was 21.29% in line with reports in the literature. We defined the pCNV yields and profiles of a Romanian cohort of patients with unexplained GDD/ID associated with different features in the context of other European studies.

Our study reinforces CMA as an effective diagnostic tool for both detection and precise characterization of clinically relevant CNVs in patients with GDD/ID, ASD and MCA. CMA can characterize the regions involved in structural abnormalities detected by conventional karyotyping. A correct and complete diagnosis dictates that CMA and conventional karyotyping should be used complementarily in certain instances. Parental analysis is essential for genetic counselling, particularly when the patient has terminal deletion/duplication or large CNVs.

The main reasons for referral for CMA testing in our study were GDD/ID, MCA, dysmorphic facial features, and ASD. Dysmorphic facial features and ASD (as a main or secondary feature) and secondary phenotypes such as micro/macrocephaly, MCA, psychiatric disorders, ADHD or speech/language delay are possible predictive phenotypes of a higher diagnostic rate through CMA.

Due to its wide application and clear cost effectiveness, CMA is now the most efficient cytogenetic screening method routinely used in genetic diagnostics and it is only likely to be replaced by when the costs of NGS based methods are significantly reduced.

## Figures and Tables

**Table 1:** pCNVs identified in our cohort. pCNVs found by CMA in the cohort, with the number of OMIM genes present in the region, listing the phenotypes for each individual.

Patient # /Gender	pCNVs Identified	pCNVs Size (Mb)	No. of OMIM Genes	Phenotype	Diagnosed Syndrome	*De novo* Status
#2 M	arr[hg19]7q11.23 (72, 766, 313-74, 133, 332) ×1	1.36 Mb	24	MID, CA, FD	Williams–Beuren syndrome (OMIM 194050; ORPHA 904)	*de novo*
#7 M	arr[hg19]3q13.2-q13.31 (112, 183, 943-115, 492, 949) ×1	3.30 Mb	18	GDD/MID, ASD, SLD, FD		ND
#11 M	arr[hg19]9p24.2-p22.3 (4, 382, 484-16, 182, 060) ×1	11.80 Mb	34	GDD/MID, SLD, FD		ND
#28 M	arr[hg19]15q11.2-q13.3 (22, 765, 628-32, 418, 879) ×3	9.65 Mb	35	GDD/MID, SLD, FD	15q11.2 duplication syndrome (OMIM 608636; ORPHA 238446)	*de novo*
#29 F	arr[hg19]3q28-q29 (190, 674, 919-197, 837, 049) ×3	7.16 Mb	44	MID, CA, FD		ND
	arr[hg19]18q22.3-q23 (71, 021, 353-78, 010, 032) ×1	6.98 Mb	20			ND
#38 M	arr[hg19]17q12 (34, 822, 500-36, 248, 918) ×1	1.42 Mb	14	PID, SLD	17q12 deletion syndrome (OMIM 614527; ORPHA 261265)	ND
#39 M	arr[hg19]Xq28 (151, 371, 831-155, 226, 073) ×3	3.85 Mb	2	MID, SLD, CA, FD		ND
#40 F	arr[hg19]15q11.2-q13.3 (22, 469, 323-32, 432, 126) ×3	9.96 Mb	35	MID, ASD	15q11.2 duplication syndrome (OMIM 608636; ORPHA 238446)	*de novo*
#44 F	arr[hg19]1q21.3 (153, 832, 229-154, 473, 676) ×1	0.64 Mb	15	GDD/MID, CA, FD		ND
#49 M	arr[hg19]13q34 (111, 574, 034-112, 873, 904) ×1	1.30 Mb	2	MID, SLD, FD		ND
#54 M	arr[hg19]17p11.2 (16, 782, 546 -20, 294, 038) ×1	3.51 Mb	41	GDD/MID, ASD, SLD, CA, FD	Smith–Magenis syndrome (OMIM 182290; ORPHA 819)	*de novo*
#56 M	arr[hg19]15q13.3 (31, 972, 646-32, 509, 926) ×3	0.53 Mb	2	GDD/MID, SLD, CA, FD		*de novo*
#60 F	arr[hg19]7q36.1-q36.3 (148, 039, 892-159, 125, 464) ×1	11.00 Mb	71	GDD/MID, SLD, CA, FD		ND
#63 F	arr[hg19]10p12.31-p11.22 (19, 126, 070-32, 661, 401) ×3	13.50 Mb	47	GDD/MID, ASD, FD		ND
#65 F	arr[hg19]11q23.3-q24.3 (118, 633, 886-134, 934, 196) ×1	16.30 Mb	102	GDD/SID, SLD, CA, FD		ND
#71 F	arr[hg19]8p23.3-p21.1 (191, 530-27, 794, 516) ×3	27.60 Mb	130	MID		ND
#72 M	arr[hg19]7p14.2-p11.2 (36, 087, 852-54, 131, 443) ×1	18.04 Mb	66	MID, SLD, CA	Greig cephalopolysyndactyly contiguous gene syndrome (OMIM 175700; ORPHA 380)	*de novo*
#74 M	arr[hg19]15q13.3 (32, 065, 000-32, 443, 078) ×3	0.37 Mb	2	GDD/SID, ASD		*de novo*
#77 M	arr[hg19]22q13.1-q13.33 (40, 731, 210-51, 178, 264) ×3	10.44 Mb	107	GDD/MID, SLD, CA, FD		*de novo*
#90 M	arr[hg19]12q15-q21.2 (69, 970, 372-77, 106, 446) ×1	7.10 Mb	27	GDD/MID, SLD, CA, FD		ND
#95 F	arr[hg19]3p26.3 (2, 300, 379-2, 371, 253) ×1	0.07 Mb	1	GDD/MID, SLD, CA, FD		ND
#96 F	arr[hg19]18p11.32-p11.21 (148, 963-14, 081, 887) ×1	13.93 Mb	56	GDD/MID, SLD, CA, FD		*de novo*
#102 M	arr[hg19]20p12.1 (15, 849, 333-17, 190, 245) ×3	1.34 Mb	4	MID, SLD, FD		ND
#107 M	arr[hg19]2q13 (110, 457, 697-111, 103, 309) ×3	0.64 Mb	5	MID, ASD, SLD, FD		ND
#112 M	arr[hg19]15q13.2-q13.3 (30, 954, 726-32, 509, 926) ×1	1.55 Mb	6	GDD/MID, ASD, SLD, FD		*de novo*
#118 F	arr[hg19]8p23.3-p23.1 (191, 530-10, 724, 642) ×1	10.53 Mb	32	GDD/MID		ND
	arr[hg19]15q26.1-q26.3 (92, 055, 381-102, 383, 473) ×3	10.32 Mb	21			*de novo*
#122 F	arr[hg19]22q13.2-q13.33 (43, 072, 344-51, 178, 264) ×1	8.10 Mb	65	GDD/SID, FD	Phelan–McDermid syndrome (OMIM 606232; ORPHA 48652)	*de novo*
#124 M	arr[hg19]15q11.2 (25, 520, 851-25, 610, 995) ×1	0.09 Mb	2	GDD/MID, SLD, FD	Prader–Willi/Angelman/ 15q11.2 deletion syndrome (OMIM 176270/105830/615656; ORPHA 739/72/261183)	*de novo*
#129 F	arr[hg19]Xp11.23-p11.22 (48, 204, 101-52, 613, 025) ×3	4.40 Mb	74	MID		ND
#131 F	arr[hg19]18p11.32-p11.21 (148, 963-14, 081, 887) ×1	13.90 Mb	56	GDD/MID		ND
#134 M	arr[hg19]22q11.1-q11.21 (17, 397, 498-18, 628, 078) ×3	1.23 Mb	10	MID, SLD, CA, FD	22q11.2 duplication syndrome (OMIM 608363; ORPHA 1727)	*de novo*
#137 M	arr[hg19]22q11.21 (18, 894, 835-21, 505, 417) ×3	2.61 Mb	44	MID, ASD, SLD, FD	22q11.2 duplication syndrome (OMIM 608363; ORPHA 1727)	*de novo*
#140 M	arr[hg19]2q13 (110, 862, 477-110, 964, 737) ×1	0.10 Mb	2	MID, ASD, SLD, FD		ND
#141 M	arr[hg19]5q23.1 (116, 416, 138-120, 031, 429) ×3	3.61 Mb	5	GDD/MID		ND
#143 F	arr[hg19]15q11.2 (22, 765, 628-23, 300, 287) ×1	0.53 Mb	4	GDD/MID, FD	Prader–Willi/Angelman/ 15q11.2 deletion syndrome (OMIM 176270/105830/615656; ORPHA 739/72/261183)	*de novo*
#153 M	arr[hg19]13q31.2-q32.2 (88, 267, 238-98, 888, 611) ×1	10.62 Mb	25	MID, CA, FD		ND
#156 M	arr[hg19]15q11.2-q13.1 (22, 833, 122-28, 691, 460) ×3	5.85 Mb	24	GDD/MID, ASD, SLD, CA, FD	15q11.2 duplication syndrome (OMIM 608636; ORPHA 238446)	*de novo*
#165 M	arr[hg19]15q11.2-q13.1 (22, 765, 628-28, 691, 460) ×3	5.92 Mb	24	GDD/MID, FD	15q11.2 duplication syndrome (OMIM 608636; ORPHA 238446)	*de novo*
#176 M	arr[hg19]9p13.1-p11.2 (39, 254, 329-43, 704, 969) ×1	4.45 Mb	4	MID, SLD		ND
#177 F	arr[hg19]3p21.31 (49, 067, 306-49, 348, 838) ×3	0.28 Mb	7	GDD/MID, CA, FD		ND
	arr[hg19]Xp22.31 (6, 488, 721-8, 097, 511) ×3	1.60 Mb	4			ND
#178 M	arr[hg19]8q24.11-q24.13 (118, 411, 534-125, 872, 913) ×1	7.46 Mb	28	MID, FD		ND
#187 M	arr[hg19]15q11.1-q13.3 (20, 686, 203-32, 631, 681)×3	11.95 Mb	37	GDD/MID, FD	15q11.2 duplication syndrome (OMIM 608636; ORPHA 238446)	*de novo*
#188 M	arr[hg19]1q21.1-q21.2 (145, 899, 359-147, 824, 212) ×3	1.92 Mb	13	GDD, FD	1q21.1 duplication syndrome (OMIM 612475; ORPHA 250994)	ND
#190 F	arr[hg19]16p12.2 (21, 444, 452-21, 926, 420) ×1	0.48 Mb	3	GDD/SID, SLD, FD	16p12.2 deletion syndrome (OMIM 613604; ORPHA 261211)	*de novo*
#194 M	arr[hg19]7q35 (145, 868, 726-145, 971, 078) ×1	0.10 Mb	1	GDD/MID, ASD, SLD, FD		ND
#205 M	arr[hg19]16p11.2-p11.1 (32, 573, 813-34, 727, 365) ×3	2.15 Mb	1	GDD/PID, SLD, CA	16p11.2 duplication syndrome (OMIM 614671; ORPHA 370079)	*de novo*
#208 M	arr[hg19]19q13.2-q13.31 (43, 242, 811-43, 741, 714) ×3	0.49 Mb	10	MID, SLD, FD		ND
#214 F	arr[hg19]3p14.2-p14.1 (62, 145, 868-66, 369, 539) ×1	4.22 Mb	12	GDD/SID, SLD, CA, FD		ND
#215 F	arr[hg19] 7q11.23 (76, 139, 286-76, 557, 072) ×1	0.41 Mb	2	MID, ASD, SLD, FD	Williams–Beuren syndrome (OMIM 19405)	ND
#216 F	arr[hg19]1p36.32-p36.33 (564, 512-2, 633, 410) ×1	2.07 Mb	47	GDD/MID, FD	1p36 deletion syndrome (OMIM 607872; ORPHA 1606)	ND
#217 F	arr[hg19]2q13 (110, 862, 474-110, 964, 775) ×1	0.10 Mb	2	GDD/MID, FD		ND
#219 M	arr[hg19]1q21.1-q21.2 (146, 155, 929-147, 824, 212) ×3	1.67 Mb	13	MID, ASD, SLD, FD	1q21.1 duplication syndrome (OMIM 612475; ORPHA 250994)	ND
#220 M	arr[hg19]22q12.3 (33, 809, 250-35, 821, 348) ×3	2.01 Mb	6	GDD/MID, ASD, FD		*de novo*
#221 M	arr[hg19]2q22.2-q22.3 (142, 553, 348-144, 922, 249) ×1	2.37 Mb	4	MID, ASD		ND
#222 M	arr[hg19]2p25.2-p24.3 (6, 119, 066-23, 743, 786) ×1	17.62 Mb	50	GDD, FD		ND
#223 F	arr[hg19]17p11.2 (16, 637, 872-20, 294, 010) ×1	3.66 Mb	41	SID, ASD, SLD, FD	Smith–Magenis syndrome (OMIM 182290; ORPHA 819)	*de novo*
#225 F	arr[hg19]22q12.3 (33, 809, 250-35, 821, 348) ×3	2.01 Mb	6	GDD/MID, ASD, FD		*de novo*
#229 M	arr[hg19]14q31.3-q32.12 (89, 006, 445-93, 270, 145) ×1	4.26 Mb	23	MID, SLD, FD		ND
#230 M	arr[hg19]1q21.1-q21.2 (143, 700, 143-149, 754, 257) ×1	6.05 Mb	41	MID, ASD, SLD, FD	1q21.1 deletion syndrome (OMIM 612474; ORPHA 250989)	ND
#234 F	arr[hg19]5p15.33-p15.2 (22, 149-10, 213, 019) ×1	10.16 Mb	37	GDD/MID, SLD, CA, FD	Cri du chat syndrome (OMIM 123450; ORPHA 281)	*de novo*
	arr[hg19]15q25.2-q26.3 (84, 084, 270-102, 383, 479) ×3	18.29 Mb	71			*de novo*
#251 F	arr[hg19]9p24.3 (204, 090-318, 901) ×1	0.11 Mb	1	GDD/MID, FD		ND
#256 M	arr[hg19]18q21.31-q23 (54, 370, 373-78, 012, 819) ×1	23.55 Mb	68	GDD/SID, ASD, SLD, CA, FD	Distal 18q deletion syndrome (OMIM 601808)	ND
#258 M	arr[hg19]18q22.1-q23 (61, 916, 757-78, 012, 819) ×1	16.01 Mb	30	GDD/MID, SLD, CA, FD	Distal 18q deletion syndrome (OMIM 601808)	ND
#264 M	arr[hg19]2q31.3 (181, 725, 071-182, 872, 274) ×1	1.15 Mb	7	GDD/MID, SLD		ND
#269 M	arr[hg19]15q11.2-q13.2 (23, 707, 435-30, 366, 138) ×3	6.94 Mb	24	GDD/MID, ASD, SLD, FD	15q11.2 duplication syndrome (OMIM 608636; ORPHA 238446)	*de novo*
#275 M	arr[hg19]10q11.22 (46, 699, 438-47, 768, 540) ×3	1.07 Mb	8	GDD/MID, ASD, SLD, CA, FD		ND
#283 F	arr[hg19]7p15.2 (27, 216, 450-27, 529, 778) ×1	0.31 Mb	6	GDD/MID, SLD, FD		ND
#290 F	arr[hg19]22q11.21 (18, 765, 102-21, 661, 435) ×1	2.90 Mb	45	MID, ASD, SLD, CA, FD	DiGeorge syndrome (OMIM 188400; ORPHA 567)	*de novo*
#297 F	arr[hg19]21q11.2-q22.3 (15, 485, 038-48, 090, 352) ×3	32.61 Mb	165	GDD/MID, SLD, FD	22q11.2 duplication syndrome (OMIM 608363; ORPHA 1727)	*de novo*
#301 M	arr[hg19]13q33.2 (105, 143, 800 -106, 578, 383) ×1	1.43 Mb	2	MID, SLD		ND
#302 M	arr[hg19]3q24-q29 (142, 811, 019-197, 837, 069) ×3	55.02 Mb	228	GDD/MID, FD	3q29 duplication syndrome (OMIM 611936; ORPHA 251038)	ND
#307 F	arr[hg19]15q11.2 (22, 698, 520-23, 260, 534) ×1	0.56 Mb	4	GDD/MID, ASD, SLD, FD	Prader–Willi/Angelman/ 15q11.2 deletion syndrome (OMIM 176270/105830/615656; ORPHA 739/72/261183)	*de novo*
#315 F	arr[hg19]15q11.1-q13.1 (20, 686, 203-28, 592, 766) ×3	7.91 Mb	26	MID	15q11.2 duplication syndrome (OMIM 608636; ORPHA 238446)	*de novo*
#317 M	arr[hg19]14q32.2-q32.31 (100, 396, 820-101, 488, 898) ×1	1.09 Mb	22	MID, SLD, FD		ND
#328 M	arr[hg19]8p23.3-p23.2 (61, 749-4, 173, 771) ×1	4.11 Mb	6	GDD/MID, SLD, FD		*de novo*
	arr[hg19]8q23.3-q24.3 (117, 002, 727-146, 280, 167) ×3	29.28 Mb	137		Langer–Giedion syndrome (OMIM 150230; ORPHA 502)	*de novo*
#344 F	arr[hg19]20q13.13 (46, 786, 589-47, 852, 910) ×3	1.07 Mb	5	MID, FD		ND
#348 M	arr[hg19]15q24.1-q24.2 (73, 703, 885-75, 257, 869) ×1	1.55 Mb	29	GDD/SID, ASD, SLD, FD	15q24 deletion syndrome (OMIM 613406; ORPHA 94065)	*de novo*
#351 M	arr[hg19]Xp22.31 (6, 488, 462-7, 809, 341) ×1	1.32 Mb	2	MID, ASD, SLD, FD		ND
#370 M	arr[hg19]16p11.2 (29, 673, 967-30, 332, 569) ×1	0.65 Mb	21	GDD/MID, ASD, SLD, FD	Distal 16p11.2 deletion syndrome (OMIM 613604; ORPHA 261211)	*de novo*

Dup = Duplication, Del = Deletion, CA = congenital anomalies, GDD = global developmental delay, MID = mild/moderate intellectual disability, SID = severe intellectual disability, ASD = autism, FD = facial dysmorphisms, SLD = speech and/or language delay or impairment, ADHD = Attention-deficit/hyperactivity disorder, LD = Learning disability, ND = not determined. F = Female, M = Male.

**Table 2:** Clinical characteristics of patients with unexplained GDD/ID in our cohort and the diagnosis yield of pCNVs in cases with and without a condition. Odds ratio—OR (95% confidence intervals—CI), *p*-value presented for each feature.

Clinical Features	Patients with Condition % (*n* = 371)	Patients with pCNV and with Condition % (*n*)	Patients with pCNV and without Condition % (*n*)	OR (95% CI), *p*-Value
**Intellectual disability** **total**	**371**	**79**	**-**	
mild	34.78% (129/371)	27.85% (22/79)		
moderate	49.05% (182/371)	59.49% (47/79)		
severe	14.82% (55/371)	10.12% (8/79)		
profound	1.34% (5/371)	2.53% (2/79)		
**ASD**	36.65% (136/371)	32.91% (26/79)	67.08% (53/79)	0.81 (0.48–1.37), *p* = 0.21
**ADHD**	20.21% (75/371)	22.78% (18/79)	77.21% (61/79)	1.21 (0.66–2.21), *p* = 0.26
**Speech/language delay**	64.15% (238/371)	63.29% (50/79)	36.70% (29/79)	0.95 (0.56–1.59), *p* = 0.42
**Learning disability**	6.46% (24/371)	5.06% (4/79)	94.93% (75/79)	0.72 (0.24–2.18), *p* = 0.28
**Aggressive behavior**	10.24% (38/371)	11.39% (9/79)	88.60% (70/79)	1.16 (0.52–2.57), *p* = 0.35
**Psychiatric disturbance**	**11.05% (41/371)**	**6.32% (5/79)**	**93.67% (74/79)**	**0.48 (0.18–1.26), *p* = 0.06**
**Sleep problems**	5.12% (19/371)	3.79% (3/79)	96.20% (76/79)	0.68 (0.19–2.39), *p* = 0.27
**Eating disorder**	1.07% (4/371)	1.26% (1/79)	98.73% (78/79)	1.23 (0.12–12.03), *p* = 0.42
**Motor delay**	61.18% (227/371)	62.02% (49/79)	37.97% (30/79)	1.04 (0.62–1.74), *p* = 0.43
**Ophthalmological impairment**	16.71% (62/371)	20.25% (16/79)	79.74% (63/79)	1.35 (0.72–2.55), *p* = 0.17
**Hearing impairment**	**4.04% (15/371)**	**7.59% (6/79)**	**92.40% (73/79)**	**2.58 (0.89–7.49), *p* = 0.04**
**Facial dysmorphism**	**83.28% (309/371)**	**89.87% (71/79)**	**10.12% (8/79)**	**2.01 (0.91–4.43), *p* = 0.04**
**Congenital malformations**	**16.71% (62/371)**	**21.51% (17/79)**	**78.48% (62/79)**	**1.50 (0.80–2.80), *p* = 0.09**
**Cranial anomalies** **(microcephaly/** **macrocephaly)**	37.46% (139/371)	36.70% (29/79)	63.29% (50/79)	0.95 (0.57–1.60), *p* = 0.44
**Skeletal anomalies**	20.75% (77/371)	17.72% (14/79)	82.27% (65/79)	0.78 (0.41–1.49), *p* = 0.23
**Muscular anomalies**	20.21% (75/371)	17.72% (14/79)	82.27% (65/79)	0.81 (0.43–1.55), *p* = 0.27
**Limbs abnormalities**	12.93% (48/371)	12.65% (10/79)	87.34% (69/79)	0.97 (0.46–2.04), *p* = 0.46
**Fingers abnormalities**	**25.87% (96/371)**	**34.17% (27/79)**	**65.82% (52/79)**	**1.68 (0.98–2.87), *p* = 0.03**
**Short stature**	11.05% (41/371)	8.86% (7/79)	91.13% (72/79)	0.74 (0.31–1.73), *p* = 0.24

**Table 3:** Diagnostic yields from recent studies that used chromosomal microarrays for diagnostic testing in European cohorts of individuals diagnosed with syndromic or non-syndromic GDD/ID.

Study/Year	Cohort	CMA Platforms	Sample No.	Detection Rate of pCNVs
Di Gregorio et al., 2016 [21]	Patients with GDD/ID in Italy	Agilent 60k	1015	11.0%
Wolfe et al., 2017 [41]	Patients with ID, adults	Nimblegen 135k	202	11.0%
Quintela et al., 2017 [19]	Patients with ID from NW Spain	Cytogenetics Whole-Genome 2.7M SNP array/CytoScan High-Density SNP array	573	11.2%
Peycheva et al., 2018 [42]	Patients with epileptic seizures, DD/ID, autistic features, and additional brain malformations	Agilent 180k	92	15.2%
Miclea et al., 2019 [43]	Patients with GDD/ID and obesity in Cluj, Romania	Infinium OmniExpress-24, Illumina	36	33.0%
Wayhelova et al., 2019 [44]	Patients with GDD/ID, ASD and MCA from Czech Republic, children	Agilent 60k/180k/OGT 180k	542	5.9%

## Data Availability

Not applicable.
